# Determinants to antiretroviral treatment non-adherence among adult HIV/AIDS patients in northern Ethiopia

**DOI:** 10.1186/s12981-017-0143-1

**Published:** 2017-03-21

**Authors:** Berhe Beyene Gebrezgabher, Yigzaw Kebede, Melaku Kindie, Desalegn Tetemke, Mebrahtu Abay, Yalemzewod Assefa Gelaw

**Affiliations:** 1 Department of Public Health, Health Science College, Axum University, Axum, Ethiopia; 2 Department of Epidemiology and Biostatistics, Institute of Public Health, College of Medicine and Health Science, University of Gondar, 196 Gondar, Ethiopia

**Keywords:** Antiretroviral therapy, Case–control study, Non-adherence, Northern Ethiopia

## Abstract

**Background:**

Adhering 95% and above of antiretroviral therapy reduces the rate of disease progression and death among people’s living human immunodeficiency virus. Though manifold factors have reported as determinant factors of antiretroviral therapy adherence status, perhaps determinants of non-adherence differ up on the activities of patients in the study setting.

**Methods:**

An institution based unmatched case–control study was conducted in Aksum town. Individuals who had a 6-month follow-up with complete individual information were included in the study. Document review and interviewer based techniques were used to collect the data. Binary logistic regression analysis was used to identify the determinant factors of non-adherence.

**Results:**

A total of 411 (137 cases and 274 control) study participants were included in the study. The majority of them were male in sex. Having 2 years and above duration on ART [AOR = 7, 95% CI (2.2, 22.6)], history of adverse effect [AOR = 6.9, 95% CI (1.4, 32.9)], substance use [AOR = 5.3, 95% CI (1.4, 20.0)], living with parents [AOR = 3.4, 95% CI (1.2, 10.3)], having depression symptom [AOR = 3.3, 95% CI (1.4, 7.5)], <350 cells/mm^3^ cluster of differentiation 4 count [AOR = 3.2, 95% CI (1.8, 5.8)] and low dietary diversity [AOR = 2, 95% CI (1.1, 3.7)] were found significant determinants of non-adherence to antiretroviral drug.

**Conclusion:**

Program, social and individual related factors showed a statistically significant associated with non-adherence to antiretroviral therapy. Managing lifestyle by developing self-efficacy of individuals and treating related threat to improve adherence status of antiretroviral therapy is recommended in this study.

## Background

In 2013 globally, 35 million people living with Human Immunodeficiency Virus (HIV). Worthily, sub-Saharan Africa (23.5–26.1 million) people living with HIV which is 71% of the global prevalence UNAIDS [1]. Ethiopia, an estimated 793,700 (716,300–893,200) people living with HIV/AIDS [2].

Antiretroviral therapy (ART) is a proven treatment for HIV/ADS patients in improving the health status and quality of life of HIV/AIDS patients by reducing the rate of disease progression [3]. Appropriately taking of the treatment is the advisable option in order to obtain full benefits of ART; durable suppression of viral replication, reduced destruction of CD4 cells, prevention of viral resistance, promotion of immune reconstitution, and slowed disease progression [4]. Poor ART adherence is a notable public health problem in developing countries [5].

An individual considered as non-adherence for ART if he/she had a history of taking doses 2 or more hours before, and/or 2 or more hours after the time of a doctor’s advice to take doses or missing doses completely (i.e., <95% adherence = missing >2 doses of 30 doses or >3 doses of 60 doses) [5, 6]. In 2012, over 9.7 million people living with HIV in low- and middle-income countries were receiving ART, however, ensuring adherence to HIV treatment remains challenging in all countries [7].

Adherence of ART is a complex behavior, which is influenced by several determinants, majorly patient loss to follow-up and ensuring adherence to ART regimens remain major challenges in Ethiopia [2, 5].

Variables such as; availability of reminder, substance use, malnutrition, dietary diversity, CD4 count, depression symptom, adverse effect of ART and duration on ART were reported as a determinant factor for non-adherence to ART [5, 6, 8–12]. Duration on ART [13, 14] and CD4 count in [11, 15] were associated with non-adherence to ART. Determinant factors for non-adherence are multiple and have different effects. This study was aimed to identify determinants of non-adherence to ART among HIV-infected adults in Aksum town health facilities, northern Ethiopia.

## Methods

An institution based unmatched case–control study was carried out from March 20 to May 15, 2015, in Aksum Health Center and Aksum Hospital, Aksum, Ethiopia. Aksum town is located 1067 km away in Northeast Ethiopia of Addis Ababa, the capital city of Ethiopia.

### Sample size and sampling procedure

The sample size of the study was calculated by Epi-Info™ 7 software Statcalc program using the following assumptions; proportion of non-adherence among not exposed (controls) 12.1%, proportion of non-adherence among exposed (cases) 21.7%, odds ratio 2.18 [16], 5% level of significance, 80% power of the study and 1:2 case to control ratio.

All HIV/AIDS positive adults (18+ years old) who had at least three consecutive visits before a data collection period were considered as a study population of this study. The baseline data was collected from two health facilities using clinical record review. For the present study, study groups based on ART adherence status was defined as cases; patients who had a history of greater 5% (missing >2 doses out of 30 doses or >3 doses out of 60 doses) all of scheduled regimens which supposed to take in a month and controls were near-perfect (>95%) take to dose of all regimens which supposed to take in a month [17, 18]. All cases who had to follow-up during the data collection period were included in the study whereas controls were included by systematic random sampling techniques in every other four appointees.

### Data collection and data analysis

The data were collected using chart review and interviewer administered techniques by two diploma nurses and three case managers working in ART clinics supervised by two bachelor nurse professionals. The structured questionnaire consisted of socio-demographic, socio-economic, nutritional, medical and psychological were used for the data collection tool. A 1-day training on the objective of the study, confidentiality of information and interviewing techniques were given. Baseline information was filled out from ART registration and follow-up forms.

Dietary diversity was assessed by asking the number of reported different foods and food groups consumed in a household over a 24-h period of the data collection time, does not include food group consumed outside the home. It was categorized into three groups; low if <3 food items consumed, medium if 4–6 food items consumed and high if ≥6 food items consumed [19].

Patient Health Questionnaire (PHQ) with 9-items on which score ranges from 0 to 27 were used to assess the depression of the study participants. Individuals up on their response categorized into five categories as (no depression, mild depression, moderate depression, moderately severe depression and severe depression) [20].

Precoding and manual checking of the questionnaire were done by the principal investigator before the data entry. Data was entered using Epi-Info and exported to SPSS version 20 for further cleaning and analysis purpose. Binary logistic regression model used to identify the determinant factors. Collinearity diagnostic test was conducted using tolerance to check for co-linearity between independent variables and interaction effect. Variables in the bivariable analysis having a p value <0.2 were considered for multivariate analysis to adjust the confounders. The strength and presence of statistical association were assessed by OR, p value and 95% confidence interval (95% CI). Variables with a p value ≤0.05 were considered as statistically significant determinant factors of non-adherence to ART. Hosmer–Lemeshow goodness-of-fit test (p = 0.9289) was used to assess the fitness of the model.

## Results

A total of 411 HIV-positive individuals (137 cases and 274 controls) who had at least 6 months ART follow-up participated in the study. More than half (58.2%) of the study participants were male. The mean age ± (SD) at the time of data collection for cases and controls were 37.21 (±9.95) and 35 (±8.85) years, respectively (Table 1).

**Table 1:** Socio-demographic characteristics at the time of presentation and during data collection time of cases and controls, Aksum town, northern Ethiopia 2015

Variables	Frequency of adherence status	
	Case, n (%)	Control, n (%)
Sex		
Male	69 (50.4)	170 (62)
Female	68 (49.6)	104 (38)
Age		
15–29	23 (16.8)	54 (19.7)
30–39	60 (43.8)	127 (46.4)
40–49	35 (25.5)	74 (27.0)
≥50	19 (13.9)	19 (6.9)
Marital status		
Single	25 (18.2)	47 (17.2)
Married	64 (46.7)	128 (46.7)
Widowed	14 (10.2)	32 (11.7)
Divorced/separated	34 (24.8)	67 (24.4)
Residence		
Urban	114 (83.2)	253 (92.3)
Rural	23 (16.8)	21 (7.7)
Educational level (grade)		
No formal education	29 (21.2)	57 (20.8)
Primary education (1–8)	68 (49.6)	128 (46.7)
Secondary education (9–12)	33 (24.1)	69 (25.2)
12+	7 (5.1)	20 (7.3)
Occupation		
No occupation	21 (15.3)	42 (15.3)
Government employed	25 (18.3)	52 (19)
Business/self employed	40 (29.2)	96 (35)
Daily laborer	51 (37.2)	84 (30.7)

### Determinants of non-adherence to antiretroviral therapy

In multivariable analysis variables, such as duration of treatment, ART adverse effect, substance use, living condition, depression, CD4 cell count and dietary diversity were found to be independent determinant factors with non-adherence to ART.

Patients who had more than 2 years ART follow-up [AOR = 7, 95% CI (2.2, 22.6)] were 7 times more likely to not adhere as compared to those who had 6–12 months follow-up. Patients who had a history of ART adverse drug effect [AOR = 6.9, 95% CI (1.4, 32.9)] were 6.9 times more likely to be non-adherent as compare to the counterpart. HIV-positive substance user [AOR = 5.3, 95% CI (1.4, 20)] individuals who were 5.3 times more likely to be non-adherence as compared with the counterpart.

Non-adherence were 3.4 times more likely to occur among those who live with their parents [AOR = 3.4, 95% CI (1.2, 10.3)] as compared to those who live alone. This study also showed that depression is associated with non-adherence of ART. The odds of non-adherence were 3.3 times more likely to those who had depression symptom [AOR = 3.3, 95% CI (1.4, 7.5)] as compared to their counterparts.

HIV-positive individuals who had <350 cells/mm^3^ CD4 cell count [AOR = 3.2, 95% CI (1.8, 5.8)] were 3.2 times more likely to not adhere as compared to those HIV-positive individuals who had CD4 cell count ≥350 cells/mm^3^. Moreover, low dietary diversity [AOR = 2, 95% CI (1.1–3.7)] were two times to not adhere as compared to the counterparts (Table 2).

**Table 2:** Binary and multiple logistic regression model showing determinants of non-adherence to antiretroviral therapy, among adults on ART Aksum town, northern Ethiopia 2015

Explanatory variables	Adherence status to ART		COR (95% CI)	AOR (95% CI)
	Non-adherent number (%)	Adherent number (%)		
Sex				
Male	69 (50.4)	170 (62)	1	1
Female	68 (49.6)	104 (38)	1.6 (1.1–2.4)	1.4 (0.8–2.4)
Age				
18–29	23 (16.8)	54 (19.7)	1	1
30–39	60 (43.8)	127 (46.4)	1.1 (0.6–2.0)	1.1 (0.5–2.4)
40–49	35 (25.5)	74 (27)	1.1 (0.6–2.1)	0.7 (0.3–1.7)
≥50	19 (13.9)	19 (6.9)	2.3 (1.1–5.2)	1.7 (0.6–5.0)
Residence				
Urban	114 (83.2)	253 (92.3)	1	1
Rural	23 (16.8)	21 (76.7)	2.4 (1.3–4.6)	1.4 (0.5–4.0
Occupation				
No occupation	21 (15.3)	42 (15.3)	1	1
Employed	25 (18.3)	52 (19)	0.9 (0.5–2)	1.4 (0.5–4.2)
Business	40 (29.2)	96 (35)	0.8 (0.4–1.6)	1.8 (0.7–4.0)
Farmer	14 (10.2)	10 (3.7)	2.8 (1.1–7.4)	2.3 (0.5–10.3)
Daily laborer	37 (27)	74 (27)	1 (0.5–1.9)	1.9 (0.8–4.7)
Duration on ART (months)				
6–12	7 (5.1)	38 (13.9)	1	1
13–24	14 (10.2)	38 (13.9)	2.0 (0.7–5.5)	2 (0.5–8.8)
≥25	116 (84.7)	198 (72.2)	3.2 (1.4 7.4)	7 (2.2,22.6)**
ART adverse effect				
Yes	26 (19)	3 (1.1)	21.2 (6.3–71.3)	6.9 (1.4,32.9)**
No	111 (81)	271 (98.9)	1	1
Substance use				
Yes	21 (15.3)	5 (1.8)	9.7 (3.6–26.5)	5.3 (1.4–20.0)*
No	116 (84.7)	269 (98.2)	1	1
Live with				
Alone	33 (24.1)	83 (30.3)	1	1
Parents	18 (13.1)	21 (7.7)	2.2 (1.02–4.6)	3.4 (1.2–10.3)*
Family	86 (62.8)	170 (62)	1.3 (0.8–2.1)	1.5 (0.8–2.8)
Depression status				
Depressed	41 (29.9)	19 (6.9)	5.7 (3.2–10.4)	3.3 (1.4–7.5)*
Not depressed	96 (70.1)	255 (93.1)	1	1
CD4 cell count (cells/mm^3^)				
<350	58 (42.3)	45 (16.4)	3.7 (2.4–6.0)	3.2 (1.8–5.8)*
≥350	79 (57.7)	229 (83.6)	1	1
Dietary diversity				
Low	57 (41.6)	43 (15.7)	3.8 (2.4–6.1)	2 (1.1–3.7)*
Medium and above	80 (58.4)	231 (84.3)	1	1
Daily eating pattern				
≤2 meals	69 (50.4)	103 (37.6)	1.7 (1.1–2.6)	1.01 (0.6–1.8)
≥3 meals	68 (49.6)	171 (62.4)	1	1
Opportunistic infection				
Yes	28 (20.4)	5 (1.8)	13.8 (5.2–36.7)	3.6 (1–13.1)
No	109 (79.6)	269 (98.2)	1	1
Reminder use				
Yes	127 (92.7)	268 (97.8)	0.3 (0.1–0.8)	0.5 (0.–2.0)
No	10 (7.3)	6 (2.2)	1	1
Stigma				
Yes	17 (12.4)	10 (3.6)	3.7 (1.7–8.4)	2.8 (0.9–8.5)
No	120 (87.6)	264 (96.4)	1	1
WHO stage				
Stage I	16 (11.7)	68 (24.8)	1	1
Stage II and above	121 (88.3)	206 (75.2)	2.5 (1.4–4.5)	1.6 (0.8–3.5)
BMI				
Under weight	38 (27.7)	41 (15)	1	1
Normal	97 (70.8)	224 (81.7)	0.5 (0.3–0.8)	0.7 (0.3–1.3)
Over weight	2 (1.5)	9 (3.3)	0.2 (0.1–1.2)	0.4 (0.01–3.0)
Treatment other than ART				
Yes	41 (29.9)	40 (14.6)	2.5 (1.5–4.1)	1.4 (0.7–3.0)
No	96 (70.1)	234 (85.4)	1	1

* p < 0.05

** p < 0.001

## Discussion

ART has a proven clinical benefit in HIV-infected people by slow downing the progressing to AIDS. Incomplete adherence to the treatment leads to immune suppression and the selection drug resistant HIV [21]. Non-adherence increases HIV-related morbidity and mortality [22]. Numerous studies were conducted on the determinants of ART drug-adherence and/or non-adherence of HIV/AIDS patients in the country. However, studies signify the consequence of non-adherence is one or more factors that may have different effects consequently; the study setting, socio-demographic characteristics of the study participants, and sample size. This study showed that having 2 years and above duration on ART, depression symptoms, history of ART adverse effect, substance use, living with parents, <350 cells/mm^3^ CD4 count and low dietary diversity in the household as determinant factors for non-adherence to ART.

Coherent with the previous studies in Ethiopia [13, 23] HIV-positive participants who had more than 2 years ART follow-up were not taken ART drugs appropriately. This finding was also coherent with the Asia study conducted in Vientiane, Lao PDR [24]. Findings of Asia [25, 26] inconsistent with the present study, patients who have been on ART for short period were more likely to be non-adherent. The possible reason for the discrepancy could be these studies were included those patients who had less than 6 months’ duration on ART since ART at an early phase of treatment may have severed adverse drug effects. On top of the treatment duration of the patients, the accessibility of health facilities, living conditions, socio-demographic characteristics of the population in Asia is differ from Ethiopia which might have effect on the adherence status of the patient.

Like the report of previous studies conducted in Ethiopia [11] and in Nepal [26] in the present study, participants that had experience of adverse drug effect during their medication were more likely to non-adherence to ART. But, this study is not consistent with a systematic review of Asian developing countries in 2012 reported [27]. The possible explanation will be they already counselled on the possible side effects of the drug during their medication follow-up time.

Substance uses among patients during ART medication was also found to be the independent determinant factors of non-adherence, a finding consistent with a study done in southern Ethiopia [16], Tanzania [28] and Nepal [26].

In the present study, the living conditions are a determinant factor of non-adherence. Patients living with their families were more likely to non-adherence of ART drug as compared to those living independent from their families, consistent with previous study done in southern Ethiopia [29] inconsistent with a study done in Canada [30] and Cambodia [31]. The difference may be due to clients’ self-efficacy and attitude towards ART medication is stumpy in the study setting, even at a country level.

The presence of depressive symptoms during medication is also reported as one of determinant factor for ART drug adherence, supported with studies done in South-west Ethiopia [14, 29], South Africa [32] and Netherlands [33]. Possibly depressed patients could easily forget to take their dose while they managed the depression symptoms.

Similar to other studies conducted in Ethiopia [15, 34, 35], and in Africa [32, 36] the level of CD4 count during initiation of treatment have independent effect for adherence status. Non-adherence was higher among patients with a CD4 count <350 cells/mm^3^ compared to their counterparts. The finding is supported by studies in Ethiopia [15, 34, 35] however, a finding reported in Yirgalem Hospital, southern Ethiopia [23] was not consistent with the present study. This may be due to the previous study controlling for both WHO stages and CD4 cell count for the initiation criteria of ART.

Moreover, low dietary habits of the patient are reported as the independent determinant factors of non-adherence. This finding is supported by reports in South and North Ethiopia [15, 16] and Zambia [37].

Unlike previous studies, socio-demographic factors such as sex, age, residence, occupation, eating pattern and patient level factors; didn’t show statistically significant association with non-adherence of ART. This may be because of in this locales the barriers of non-adherence were beyond the listed variables.

Since identifications of case and controls were done based on the registration log book and individual chart review, this paper may be prone to misclassification bias. Because of the adherence status of the patient were assessed by patient self-report, the non-adherence status may underestimate. Moreover, information bias was also a problem while assessing dietary diversity, substance use and depression symptom.

## Conclusion

Having more than 2-year duration on ART follow-up, experiencing, adverse ART drug effect, substance use, living with parents, having depression symptom, CD4 cell count <350 cells/mm^3^ and low dietary diversity were all found independent determinant factors of non-adherence of ART drug. Defining the adherence status with viral load suppression and/or measured by detectable viral load during chronic care and identifying the treatment outcome of non-adherence patient is recommended for future researcher.
